# Management in times of crises: reflections on characteristics, avoiding pitfalls, and pathways out

**DOI:** 10.1007/s11846-022-00580-2

**Published:** 2022-08-19

**Authors:** Ricarda B. Bouncken, Sascha Kraus, Antonio de Lucas Ancillo

**Affiliations:** 1 Chair for Strategic Management and Organisation, University of Bayreuth, Bayreuth, Germany; 2 Faculty of Economics & Management, Free University of Bozen-Bolzano, Bolzano, Italy; 3 Economics and Business Management Department, University of Alcalá, Madrid, Spain; 4 Department of Business Management, University of Johannesburg, Johannesburg, South Africa

**Keywords:** Crisis management, COVID-19, Ukraine war, Climate change, Entrepreneurship, Innovation, M1, M10, M19, M20

## Abstract

The world is shaken by global crises that have severe implications for firms and their management. But what is characteristic of a global crisis, and why do firms and their managers often fail to deal proactively and strategically with coping strategies for crises. This introductory paper explains the characteristics of crises and important underlying management behavior. These behaviors are most important to understand because they might escalate or reduce the effects of a global crisis in firms. We also explain the challenges associated with emphasizing the local effects of crises while ignoring or downplaying their global effects. Finally, we present the papers in this special issue that provide specific answers that can help to deal with aspects of a global crisis.

## Introduction

Since the beginning of 2020, the world has been changing on a scale and with an intensity that no one could have predicted. While we see the emergence and consequences of the still ongoing COVID-19 pandemic, the Russian war in Ukraine and climate change in society and politics, we also perceive the strong disruptions of these crises for established businesses, entrepreneurship, but also for innovation and global management (e.g., Kuckertz et al. 2020; Aguinis et al. 2022). Firms not only need to deal with dramatic changes that were not anticipated but also need to maneuver with the long-term consequences of these crises strategically (e.g., Breier et al. 2021).

Obviously, the sudden challenges related to the health situation, social welfare, lockdown, and supply chain disruption of COVID-19 have had a strong negative impact on the overall economic situation, perhaps deepening some latent challenges that have their origins in the 2009 financial crisis (Sharma et al. 2022). Some industries and firms were more strongly affected by the situation while policymakers hile policymakers were eager to soon bring the economic situation back to pre-COVID levels (Galindo-Martín et al. 2021). Still coping with COVID-19, 2022 has shown another additional crisis caused by the war in Ukraine that will have ongoing and more severe problems in the future (Mbah & Wasum, 2022). These two sudden crises are accompanied by the most severe and boundaryless climate change that increasingly has strong negative influences of different kinds (e.g., heat waves, droughts, erosion).

Previous research can deliver some answers to the actual challenges for firms in crises. Especially the COVID-19 pandemic motivated research on the health care situation and virtual work. Yet, surprisingly little research has examined the characteristics and challenges of such global crises? What does crisis mean, and what are the specifics and challenges? On these critical questions, this introductory paper aims to disentangle the characteristics as well as local and global challenges and causes of innovation and entrepreneurship and to provide an overview of the studies in this special issue with regards to how they answer challenges related to these crises.

## Current crises and their comparison

Today, economies and firms face three major global crises. The most sudden and unanticipated is the COVID-19 pandemic crisis. Global and local economies were hit hard, sudden, and partly continuously by the adverse effects of the pandemic. COVID-19 has globally and dramatically influenced individual lives, exerted huge health and economic costs, and affected many firms in diverse ways (e.g., Meyer et al. 2022; Harms et al. 2021). For example, it has led to staggering unemployment and restrictions in normal life (Clark et al. 2020; Kraus et al. 2020). Interestingly, the COVID-19 pandemic has called for the willingness to show solidarity with others while accepting negative effects on self-interest (Cappelen et al. 2021).

The second and also sudden crisis is the ongoing war in Ukraine. When it started, it first truncated delivery of supply chain parts and food. In addition, the sanctions against Russia and the lowered supply of fossil energies have lowered global trade and supply. The sanctions show solidarity on the level of countries and super country arrangements, such as on the level of the EU (van Bergeijk 2022). However, the solidarity of the population does not always or fully match the actions and sanctions on the collective level initiated by governments. The crisis initially hit the Ukrainian population and specific firms in different countries. Yet, it is already visible that energy supply problems will increasingly exert several additional severe threats, for example, on industrial manufacturing and social wellbeing over the next months.

The third crisis of this time − climate change − has been ongoing, but its effects become more visible and severe every coming year (Bouncken et al. 2022). Compared to the progressive, long-term environmental crisis, the COVID-19 pandemic nuances the short-term effect of a major disruption. For example, individuals’ anxieties of contamination, of becoming ill or dying, connected to limitations health facilities initiated immediate and often dramatic actions from the side of the government that caused strong disruptions on the level of individuals and firms (Dosi and Soete 2022). Potentially, the more pressing the environmental problems, the greater the modifications of the conventional objective of economic growth towards the inclusion of metrics for sustainable development. Some research has already nuanced that climate change will demand changes in organizations and individuals’ behavior toward the development of identification processes that are in favor of environmental sustainability (Bouncken et al. 2022; Clauß et al. 2022).

Concerning the COVID-19 pandemic, the war in Ukraine, and the environmental crisis, we can disentangle a ‘local dimension’ related to the problem’s surface and a more ‘global dimension’ that includes the deeper global drivers and causes (Dosi and Soete 2022). Together, the three crises have nuanced effects that are first and foremost perceived and evaluated locally although their effects. Yet, they are far more long-term and global in the context of a highly interlinked globalized world, where boundaries to the crises are lower than many expect them (Dosi and Soete 2022).

## Definition and understanding of crises

Crises are related to strong changes that have not been anticipated and which are not short occurrences but which even become more severe over time (Pearson and Clair 1998b; Kraus et al. 2013).

Due to the uncertainties and disruptions, crises escalate fear in individuals and organizations. With this fear, individuals tend to hang on to their habits, engage strongly in the search for certainty, look for clear formal guidance, even authoritarian regimes, and cling to identity processes and social categorization that might lead to social effects such as polarization and the growth of intolerance (Dosi and Soete 2022). Hence, the fear that clings to crises causes extra challenges, reduces solidarity, and limits the success of actions taken towards coping with the crises. Accordingly, when solidarity is low and ego-interests high, there will be declining chances of reducing the escalating problems of a crisis.

Although environmental and economic crises might appear suddenly, their influences on firms in the appearance of organizational crises are usually understood via a process (Claeys and Cauberghe 2014; Pearson and Clair 1998a; Sayegh et al. 2004). Traditionally the literature understands an organizational crisis not as a sudden event but as a process that might be differentiated into stages (Lukason and Laitinen 2019) or specific cycles (Fernandez and Mazza 2014). So typically, the crisis emerges and remains over a period of time but might allow for actions to reduce it respectively (Trahms et al. 2013; Weitzel and Jonsson 1989). Yet, wrong actions or inactivity might escalate the process.

Even though a crisis relates to a process, previous research has revealed that an organizational crisis demands quick decisions of managers, mainly at the C-level or by the organization’s founders (Merendino and Sarens 2020). Often, the crisis evolves from a smoldering state, but to prevent its diffusion and escalation, managers need to look for immediate solutions (Williams et al. 2017). Research pinpoints that for organizational crises that there is not a single triggering or initial event but a long journey of misguidance that enfolds unknown paths (Coombs 2014; Pearson and Clair 1998a).

Similarly, an organizational crisis is rarely the outcome of only one factor but rather of different ones. Often, the combination of internal and external events causes an organizational crisis (MacDougall et al. 2016; Sheng and Lan 2019). There might be external causes and environmental uncertainty which become severe threats to the firm because of specific contextual conditions (Lorsch and MacIver 1989). The internal view or the important internal causes often relate to environmental maladaptation, combined with wrong decisions, conflicts, or misconducts of managers (Merendino and Sarens 2020). Hence, misconduct or managers might further escalate an organizational crisis while proper management behavior can shorten the crisis and deliver ways out of it. We reason that the following insights into misconduct in organizational crises might also provide guidance about how to work with a global crisis that affects the organization.

### Management misconduct in crises

Understanding the crisis as a process calls for managers to strategize and action toward identifying ways out of the ongoing crises. However, previous research has identified factors that reduce dealing with the crisis. The knowledge of such misconduct can help avoid those behaviors and guide pathways that better cope with global crises. In the following, we explain important causes and bring arguments on how they relate to the actual crises.

### Overconfidence

When a crisis unfolds, be it gradually or suddenly, managers show overconfidence in dealing with it. Overconfidence spoils effective decision-making as it produces less accurate or over-optimistic evaluations of a decision’s outcomes (Simon and Shrader 2012). In situations of overconfidence, managers’ cognitive processes are too lowly activated as they do not see the challenges or threats of the crises. In detail, overconfident managers are largely ignorant of potential problems, so they do not enter search processes, crisis planning, and the development of creative visions out of the crises (Merendino and Sarens 2020).

All the recent three crises might have been triggering overconfidence in managers. At the beginning of the pandemic, managers had not considered the dramatic and worldwide challenges that affected more and more firms in different industries and the international transfer of goods that limited or blocked the global supply chain processes. Then managers might have considered local effects but only gradually understood the severity of the global challenges. Due to the greater attention on local challenges, managers’ overconfidence lingered over a longer period. For example, the ongoing lockdowns, health issues, and virtual work relationships brought about several shortages of human resources, physical resources, and market demands. Managers largely ignored the scale and scope of these challenges.

The overconfidence and emphasis on local challenges seems to have parallels in its effects as the war in Ukraine. We propose that the energy and food supply shortages will even enfold more in the future. Yet, with the effects of the pandemic still lingering (e.g., shortage of Solar Panels), there are limited ways out of the energy shortage.

While the pandemic and the war occurred more suddenly, the climate change crisis is still evolving. Even Government policies (see Paris climate agreement) seem to have been overheard or downplayed by managers. Managers also seem to underestimate the necessary moral and identification changes needed to cope with the climate changes. In addition, climate change has very strong global and long-term effects. The typical focus on local challenges and overconfidence in mastering long-term and global problems worsen the inactivity. Overconfidence and managers’ ignorance might worsen the situation because alternative paths that demand long-term investments out of the crises have not been taken, for example, by fostering technology and necessary organizational innovation that could make an organization more sustainable.

### Gaps of knowledge and experience

Previous research reveals that managers are often not cognitively equipped to sense the seeds related to the crisis because they lack knowledge and experience for perceiving and are crucial for the global and long-term effects of the global crises. The two sudden crises today, the pandemic and the war, indeed show situations in which managers naturally lacked knowledge and experience. The most significant gaps in knowledge and experiences were associated with the pandemic. Hence, guesswork and gut feeling essentially needed to substitute similar problem solutions from the past for management during the pandemic. A bit different are the effects related to the war. Managers might have experience and knowledge in dealing with areas affected by war or where their supply chain partners are located in an area affected by war. Yet, while there have been wars constantly in the world, the Ukrainian war is different because it involves a major nuclear player and because Ukraine was a powerful supplier of food and industry parts. Yet, changes associated with uncertainty might be smaller than those of the pandemic.

A different matter is the slowly progressing climate change. While there are numerous models of how climate change plays out and who will be affected in which ways, we see great ignorance and overconfidence so that managers do not use their potentially useful experiences and knowledge. Firms might use much more of their established innovation search and implementation routines to find ways to reduce energy consumption, reduce pollution, etc. Yet, the low financial and economic incentives and the often insufficient levels of environmental solidarity limit those options. In addition, there are organizations that take advantage of holes in environmental regulations and routines, and the insolidarity might motivate other organizations to care less about climate change for now.

### Lack of independent thinking

Previous research shows that managers’ insufficient independent thinking slowed the development of creative ideas, missing proactivity, and risk-taking (Merendino and Sarens 2020). The lack of formal independence is defined as managers’ ability “to make strategic decisions consistent with the concerns and evaluations of a broader set of stakeholders” (Rindova 1999, p. 966). Interestingly, managers might be formally independent but prioritize their personal interests over the firm’s (Merendino and Sarens 2020). Hence, a lack of independence defines that even when managers can recognize and understand the seeds of a crisis, they do not react at all or sufficiently because they prioritize their personal interests (Brown et al. 2020). For example, they ignore signs in the environment about the development and increase of a crisis, and the cognitive processes about solutions and the subsequent severe changes in the firm will not be activated (Merendino and Sarens 2020). However, the COVID-19 pandemic has demanded increasing solidarity. Still, in some countries, this crisis has initiated extensive discussions about the government’s role in implementing policies that reduce economic inequality and better protect health (Cappelen et al. 2021). Hence there is an interesting ambivalence.

On the one hand, the existence of a common enemy – the virus – might bring individuals together. On the other hand, high levels of stress and fear can activate an individual’s or group’s selfish impulses. Researchers claim that the pandemic has made salient the selfless behavior of some individuals and groups while also showing the heroism of others, such as health workers (Brandt et al. 2020). People-to-people solidarity has been witnessed, such as assisting elderly people through voluntary shopping services and volunteering in health care institutions (Cappelen et al. 2021). Selfish behavior became visible by hoarding and individuals not respecting the need for social distancing (Cappelen et al. 2021).

Related to a lack of independent thinking, managers but also boards or organizations might be subject to short-termism. On the individual level, short-termism shapes a constraint as a single manager might prioritize temporal focus towards the short term and conservatism, and this often or more strongly occurs during times of great uncertainty (Flint 1999; Merendino and Sarens 2020 showed that boards prioritized short-term solutions while making decisions to enhance immediate results. Especially the great uncertainties related to the pandemic might have needed short-term and immediate solutions. Yet, the war and the climate crises should motivate long-term visions, scenarios, and strategies to cope with the challenges. Perhaps, increasingly occurring threats of climate change might trigger perceptions of (localized) challenges and drive greater levels of solidarity that are needed to cope with the global climate crisis.

### Complexity and its underestimation

Global crises are complex, especially in the negative effects they incur over time. Typically, the greater a crisis becomes, the more complex the influences and the more difficult it becomes to find ways out of the crisis.

Generally, there are unclear relationships and hard to estimate causes and effects when complexity is extremely high. While the complexity adds a threat of its own, managers might even underestimate the complexity of crises. In this, managers tend to pursue too simple actions that cannot cope with the multifaceted and fuzziness of the crises (Henning Reschke et al., 2010). In addition, in underestimating the complexity, managers tend to postpone crucial decisions, and this either ignorance or underestimation becomes more severe when the seeds of a crisis become more evident step by step (Merendino and Sarens 2020). The complexity of the pandemic became greater and greater over the course of the pandemic. However, some managers chose the right focus and proactively implemented solutions. Yet most firms and governments were confronted with unanticipated multifaceted challenges. In dealing with the pandemic, the Government initiated actions that increased the complexity for firms. Potentially, the current War bears fewer complex issues but demands hardly achievable solutions, for example, a shortage of energy supply. The severe global effects of climate change and many managers seem to underestimate the complexity. With every week passed, the magnitude of climate change increases, and so does the complexity and difficulty of finding ways out of it. Still, the crises might stimulate proactive, risk-taking, and innovative behavior of individuals and organizations towards innovation and entrepreneurship that reduces the challenges (Covin et al. 2020; Hughes et al. 2018).

### A glimpse of how crises affect entrepreneurship and innovation

While we experience a slowing down of economic factors, additionally, it has been shown that the pandemic has negatively affected entrepreneurship (Galindo-Martín et al. 2021). One might believe that a crisis mobilizes creativity and entrepreneurship (Peris-Ortiz et al. 2014; Dejardin et al. 2022). Still, these positive triggers have mostly been related to vaccination development, testing services, virtual work delivery services, and home and garden solutions. Negative effects of entrepreneurship, in general, have been put forward, stating lowered demand imposed by confinements and other limitations on mobility (Galindo-Martín et al. 2021). Accordingly, El-Erian (2010) argued that firms have been less apt to take risks during the financial crisis. Risk-taking is a precondition to innovation and entrepreneurship (Bouncken et al. 2014). In addition, firms were less open to investments in innovative firms as they evaluated the risks as too high in terms of capital loss. In particular, it has been shown that the great financial crisis limited the emergence of a dominant design. The crisis developed fewer innovations, especially a lower amount of disruptive ones (Brem et al. 2020). The COVID-19 crisis again showed different levels of creativity in dealing with the crisis, often only at a medium innovative level but technology-facilitated (Wendt et al. 2021). Just this − technology − could be the trigger for turning crises into opportunities for entrepreneurship and innovation.

Different views on a problem might also trigger innovation and entrepreneurship (Lv et al. 2021). In this vein, researchers have remarked that a crisis can bring people into a moral conundrum, which might facilitate the development and articulation of opposing intuitions and perspectives (Kluger 2020). Hence, diverse needs and opposing views related to the emergence of a global crisis might stimulate diverse thoughts over this course of entrepreneurship. However, perhaps the digitalization, artificial intelligence, the solidarity of family firms, specific business incubators, entrepreneurial strategies, new forms of knowledge work, novel shared value systems, and new digital platforms might reveal solutions to the crises and build up resilience (Hillmann 2021).

Still, one might argue that especially the issues related to climate change will encourage innovation and entrepreneurship in the form of technological progress. Including metrics for sustainable development might stimulate innovation and entrepreneurship toward new business opportunities for dealing with the new metrics (Galindo-Martín et al. 2021).

## Overview of the papers

This special issue includes eight contributions on specific solutions to crises, offering a wide overview of strategies from different perspectives. Two contributions examine business models (AI business model innovation, create shared value). Three papers investigate knowledge and information transfer (information asymmetries, knowledge worker, and email marketing). And three contributions draw on more contextual perspectives and topics, studying innovation ecosystems, business incubators, and notions in entrepreneurial strategies.

Åström et al. (2022) provide a work on “*Value creation and value capture for AI business model innovation: a threephase process framework”*. They explain how AI providers integrate value-creation and value-capture elements to develop commercially viable AI business models. Against the growing adoption of AI technology, this study used an in-depth single-case study to understand firms’ AI business model innovation toward commercializing the value of the technology. They identified three stages AI suppliers must go through: identifying AI value creation prerequisites, matching value capture methods, and establishing an AI business model. They also suggest that AI providers must develop and experiment with a wide range of AI business models to achieve economic success.

Camisón et al. (2022), in their article on “*Asset tangibility, information asymmetries and intangibles as determinants of family firms leverage”* examine the capital structure decision in small and medium-sized family firms. Drawing on a mix of theories: agency theory, the behavioral theory of the firm, and strategic theory, they look at how family control, publicly available information and tangibility affect financial structure based on survey data from 543 companies in the Spanish tourism industry. Their findings show that family ownership and leverage have a more complicated and variable relationship than conventional financial and behavioral models suggest. Their empirical evidence reveals that the relationship between family ownership and leverage capacity is also contingent on information transmission strategies, asset investment decisions, and ownership arrangements on debt capacity.

Deyanova et al. (2022), in “*Hatching startups for sustainable growth: a bibliometric review on business incubators”* conduct bibliometric performance analyses and science mappings to present a systematic overview of the scholarly knowledge base relating to business incubators. Their findings are based on reviewing and analyzing relationships between 194 published articles in this field. The performance analyses show the literature and publishing practice of researchers and organizations, including the characteristics of publications and their citations over time. The science mappings comprise keyword co-occurrence analysis and bibliographic coupling, which identify leading research topics in this field, uncovering its framework and dynamics.

In their paper entitled *“Examining subjective career success of knowledge workers”*, Gaile et al. (2022) suggest a novel model for evaluating career success by considering universal personal values, behavioral factors, and sociodemographic factors. On the fundament of theoretical implications suggested by protean career orientation, they collected data from 384 specialists and managers in Latvian organizations across 20 different industries. The results show that self-direction and power are the most impactful personal qualities on subjective career success. Even though some career behaviors, including confidence behaviors and attitudes toward incentives and relationships, significantly contribute to subjective job success, curiosity habits and educational levels present negative influences. Besides, gender shows no significant impact on their perceived professional success.

Rapp (2022) on “*Predictive vs. nonpredictive entrepreneurial strategies: What’s the difference, anyway?”* offers a novel perspective on the role of prediction and adaption in entrepreneurial action. The author argues that the two essential conceptions of how entrepreneurial activity could progress, one being the predictive approach (causation) and the other being the non-predictive or adaptive method (effectuation), should be unified rather than distinct and mutually exclusive. Through reasoning the overlaps and concurrency of the two features, this research suggests that prediction and adaptation invariably co-occur, proposing a judgment-based unified idea of predictive-adaptive entrepreneurial activity.

Rubio-Andrés et al. (2022) offer a piece on “*Driving innovation management to create shared value and sustainable growth a metric and governance model for evaluating the advantages of creating shared value in small and medium-sized enterprises”*. They draw on current discussions on the importance of creating shared value for SMEs and collected data from 1136 Spanish SMEs. This study suggests innovation management as a critical driver for creating shared value through cultural transformation processes. Furthermore, the findings also show that creative businesses may promote the development of social and economic value with reputation mediating the relationship. They propose a strategic management model to achieve shared value and sustainable growth considering innovation management, social value, and reputation.

The article *“Gaming innovation ecosystem: actors, roles, and co-innovation processes”* by Klimas and Czakon (2022) looks into innovation ecosystems and the co-innovation process among various actors. They conducted a longitudinal investigation into the Polish gaming innovation ecosystem over three years. The findings identify four co-creation roles: direct value creation, supporting value creation, encouraging entrepreneurship, and leadership. This study further suggests a five-stage co-innovation process: co-discovery, co-development, co-deployment, co-delivery, and co-dissemination.

## Data Availability

Our manuscript has no associate data.

## References

[CR1] Aguinis H, Jensen SH, Kraus S (2022) ‘Policy implications of organizational behavior and human resource management research’, *Academy of Management Perspectives*, 36(3): 1–22

[CR2] Åström J, Reim W, Parida V (2022) ‘Value creation and value capture for AI business model innovation: A three-phase process framework’,Review of Managerial Science, 10.1007/s11846-022-00521-z

[CR3] Bouncken RB, Cesinger B, Kraus S (2014). ’The role of entrepreneurial risks in the intercultural context: a study of MBA students in four nations’. Eur J Int Manage.

[CR4] Bouncken RB, Lapidus A, Qui Y (2022). ’Organizational sustainability identity:‘New Work’of home offices and coworking spaces as facilitators’. Sustainable Technol Entrepreneurship.

[CR5] Brandt A, Chan SM, Dewar M, DiMari C, Koch SA, Johns FM (2020) The heroism of health workers in the coronavirus crisis, *The New York Times*

[CR6] Breier M, Kallmuenzer A, Clauss T, Gast J, Kraus S, Tiberius V (2021) ‘The role of business model innovation in the hospitality industry during the COVID-19 crisis’, *International Journal of Hospitality Management*, 92: 102723 10.1016/j.ijhm.2020.102723 PMC9998104 36919038

[CR7] Brem A, Nylund P, Viardot E (2020). ’The impact of the 2008 financial crisis on innovation: A dominant design perspective’. J Bus Res.

[CR8] Brown R, Rocha A, Cowling M (2020). ’Financing entrepreneurship in times of crisis: Exploring the impact of COVID-19 on the market for entrepreneurial finance in the United Kingdom’. Int Small Bus Journal: Researching Entrepreneurship.

[CR9] Camisón C, Clemente JA, Camisón-Haba S (2022) ‘Asset tangibility, information asymmetries and intangibles as determinants of family firms leverage’,Review of Managerial Science, 10.1007/s11846-022-00522-y.

[CR10] Cappelen AW, Falch R, Sørensen E, Tungodden B (2021). ’Solidarity and fairness in times of crisis’. J Econ Behav Organ.

[CR11] Claeys A-S, Cauberghe V (2014). ’What makes crisis response strategies work? The impact of crisis involvement and message framing’. J Bus Res.

[CR12] Clark C, Davila A, Regis M, Kraus S (2020). ’Predictors of COVID-19 voluntary compliance behaviors: An international investigation’. Global Transitions.

[CR13] Clauß T, Kraus S, Jones P (2022) ‘Sustainability in family business: Mechanisms, technologies and business models for achieving economic prosperity, environmental quality and social equity’, *Technological Forecasting Social Change*, 176: 121450

[CR14] Coombs WT, Coombs WT (2014). A need for more crisis management knowledge.. Ongoing crisis communication.

[CR15] Covin JG, Rigtering J, Hughes M, Kraus S, Cheng C-F, Bouncken R (2020). ’Individual and team entrepreneurial orientation: Scale development and configurations for success’. J Bus Res.

[CR16] Dejardin M, Raposo ML, Ferreira JJ, Fernandes CI, Veiga PM, Farinha L (2022) ‘The impact of dynamic capabilities on SME performance during COVID-19’,Review of Managerial Science, 10.1007/s11846-022-00569-x

[CR17] Deyanova K, Brehmer N, Lapidus A, Tiberius V, Walsh S (2022) ‘Hatching start-ups for sustainable growth: A bibliometric review on business incubators’,Review of Managerial Science, 10.1007/s11846-022-00525-9

[CR18] Dosi G, Soete L (2022) ‘On the syndemic nature of crises: A Freeman perspective’, *Research Policy*, 51(1): 104393 10.1016/j.respol.2021.104393 PMC8511438 34658456

[CR19] Fernandez AL, Mazza C (2014) Boards under crisis: Board action under pressure. Springer

[CR20] Flint DH (1999). ’The role of organizational justice in multi-source performance appraisal: Theory-based applications and directions for research’. Hum Resource Manage Rev.

[CR21] Gaile A, Baumane-Vītoliņa I, Kivipõld K, Stibe A (2022) ‘Examining subjective career success of knowledge workers’,Review of Managerial Science, 10.1007/s11846-022-00523-x

[CR22] Galindo-Martín M-Á, Castaño-Martínez M-S, Méndez-Picazo M-T (2021). ’Effects of the pandemic crisis on entrepreneurship and sustainable development’. J Bus Res.

[CR23] Harms R, Alfert C, Cheng C-F, Kraus S (2021) ‘Effectuation and causation configurations for business model innovation: Addressing COVID-19 in the gastronomy industry’, *International Journal of Hospitality Management*, Vol. 95: 102896 10.1016/j.ijhm.2021.102896 PMC9616625 36341083

[CR24] Henning Reschke C, Bogenhold D, Kraus S ‘How innovation and entrepreneurship can conquer uncertainty and complexity: learning about the unexpected’, Int J of Complexity in Leadership and Management (2010) Vol. 1, No. 1, pp.55–71

[CR25] Hillmann J (2021). ’Disciplines of organizational resilience: contributions, critiques, and future research avenues’. Review of Managerial Science.

[CR26] Hughes M, Filser M, Harms R, Kraus S, Chang M-L, Cheng C-F (2018). ’Family firm configurations for high performance: The role of entrepreneurship and ambidexterity’. Br J Manag.

[CR27] Klimas P, Czakon W (2022) ‘Gaming innovation ecosystem: actors, roles and co-innovation processes’, *Review of Managerial Science*, 10.1007/s11846-022-00518-8

[CR28] Kluger J (2020) The Moral Dilemma of Coronavirus Quarantines

[CR29] Kraus S, Clauss T, Breier M, Gast J, Zardini A, Tiberius V (2020) ‘The economics of COVID-19: initial empirical evidence on how family firms in five European countries cope with the corona crisis’,International Journal of Entrepreneurial Behavior & Research, 26(5): 967–1092

[CR30] Kraus S, Moog P, Schlepphorst S, Raich M (2013) ‘Crisis and turnaround management in SMEs: a qualitative-empirical investigation of 30 companies’, 5(4): 406–430

[CR31] Kuckertz A, Brändle L, Gaudig A, Hinderer S, Morales Reyes CA, Prochotta A, Steinbrink KM, Berger ESC (2020) ‘Startups in times of crisis – A rapid response to the COVID-19 pandemic’, *Journal of Business Venturing Insights*, 13: e00169

[CR32] Cappelen, A. W., Falch, R., Sørensen, E. Ø., & Tungodden, B. (2021). Solidarity and fairness in times of crisis. Journal of Economic Behavior & Organization, 186, 1–11. 10.1016/j.jebo.2021.03.017 PMC9754792 36540059

[CR33] Lorsch J, MacIver E (1989) *The reality of America’s corporate board*, Cambridge, MA

[CR34] Lukason O, Laitinen EK (2019). ’Firm failure processes and components of failure risk: An analysis of European bankrupt firms’. J Bus Res.

[CR35] Lv Z, Rodríguez-García M, Sendra-García J (2021). ’Does institutional quality affect the level of entrepreneurial success differently across the entrepreneurship distribution?‘. Rev Man Science.

[CR36] MacDougall A, Ritchie L, Yalden R, Bradley N (2016) The Board’s Role in Crisis Management.In Osler, Hoskin & Harcourt (editor), Toronto, 20-22.

[CR37] Mbah RE, Wasum D, .R.J (2022) ‘Russian-Ukraine 2022 War: A review of the economic impact of Russian-Ukraine crisis on the USA, UK, Canada, and Europe’, Advances in Social Sciences Research, 9(3): 144–153

[CR38] Merendino A, Sarens G (2020). ’Crisis? What crisis? Exploring the cognitive constraints on boards of directors in times of uncertainty’. J Bus Res.

[CR39] Meyer N, Niemand T, Davila A, Kraus SJPo (2022) ‘Biting the bullet: When self-efficacy mediates the stressful effects of COVID-19 beliefs’, PLoS One, 17(1): e0263022 10.1371/journal.pone.0263022 PMC8797252 35089967

[CR40] Pearson CM, Clair JA (1998). ’Reframing Crisis Management’. Acad Manage Rev.

[CR41] Pearson CM, Clair JA (1998b) J.A.o.m.r. ‘Reframing crisis management’, 23(1):59–76

[CR42] Peris-Ortiz M, Fuster-Estruch V, Devece-Carañana C (2014) Entrepreneurship and innovation in a context of crisis. Entrepreneurship, innovation and economic crisis. Springer, pp 1–10

[CR43] Rapp DJ (2022) ‘Predictive vs. non-predictive entrepreneurial strategies: What’s the difference, anyway?‘,Review of Managerial Science, 10.1007/s11846-022-00519-7

[CR44] Rindova VP (1999). ’What corporate boards have to do with strategy: A cognitive perspective’. J Manage Stud.

[CR45] Rubio-Andrés M, del Mar Ramos-González M, Sastre-Castillo M (2022) ‘Driving innovation management to create shared value and sustainable growth’,Review of Managerial Science, 10.1007/s11846-022-00520-0

[CR46] Sayegh L, Anthony WP, Perrewé PL (2004). ’Managerial decision-making under crisis: The role of emotion in an intuitive decision process’. Hum Resource Manage Rev.

[CR47] Sharma GD, Kraus S, Liguori E, Bamel UK, Chopra R (2022) ‘Entrepreneurial Challenges of COVID-19: Re-thinking entrepreneurship after the crisis’,Journal of Small Business Management, 10.1080/00472778.2022.2089676

[CR48] Sheng J, Lan H (2019). ’Business failure and mass media: An analysis of media exposure in the context of delisting event’. J Bus Res.

[CR49] Simon M, Shrader R (2012) ‘Entrepreneurial actions and optimistic overconfidence: The role of motivated reasoning in new product introductions’, 27(3): 291–309

[CR50] Trahms CA, Ndofor HA, Sirmon DG (2013). ’Organizational decline and turnaround: A review and agenda for future research’. J Manag.

[CR51] van Bergeijk PA (2022) ‘Sanctions against the Russian war on Ukraine: Lessons from history and current prospects’, Journal of World Trade, 56(4)

[CR52] Weitzel W, Jonsson E (1989). ’Decline in Organizations - a Literature Integration and Extension’. Adm Sci Q.

[CR53] Wendt C, Adam M, Benlian A, Kraus S, .F (2021) ‘Let’s connect to keep the distance: How SMEs leverage information and communication technologies to address the COVID-19 crisis’, Inf Syst Front, 10.1007/s10796-021-10210-z 10.1007/s10796-021-10210-z PMC8510881 34658661

[CR54] Williams TA, Gruber DA, Sutcliffe KM, Shepherd DA, Zhao EY (2017). ’Organizational response to adversity: Fusing crisis management and resilience research streams’. Acad Manag Ann.

